# Homocysteine Is a Marker of Increased Cardio-Cerebrovascular Disease Risk in Psoriatic Patients, but It Does Not Reflect the Effect of Biological Therapy in the Longitudinal Observation

**DOI:** 10.1155/2022/3820094

**Published:** 2022-01-31

**Authors:** Janette Baloghova, Eva Feketeova, Peter Kolarcik

**Affiliations:** ^1^Department of Dermatovenerology, Faculty of Medicine, P.J. Šafárik University and Louis Pasteur University Hospital, 04011 Košice, Slovakia; ^2^Department of Neurology, Faculty of Medicine, P.J. Šafárik University and Louis Pasteur University Hospital, 04011 Košice, Slovakia; ^3^Department of Health Psychology and Research Methodology, Faculty of Medicine, P.J. Šafárik University, 04011 Košice, Slovakia

## Abstract

**Background:**

Psoriasis is linked to atherosclerosis. Homocysteine (HCYS) has been identified as a marker of increased risk of cardio-cerebrovascular diseases (CCVD) in population.

**Objective:**

The aim of the study was to determine whether elevated HCYS serves as a marker of increased CCVD in psoriasis and whether biological therapy for long-term monitoring influences HCYS levels.

**Methods:**

Clinical data, laboratory tests, and comorbid diagnoses were summarized for the two groups of patients based on entrance HCYS levels. Patients (*n* = 76) were included in the follow-up gradually over a period of 5 years.

**Results:**

The psoriatic patients with normal (54%) and elevated (46%) HCYS before biological treatment did not vary in clinical data, laboratory tests, treatment, and comorbid diagnoses apart from CCVD. Elevated HCYS group showed a four-fold excess of CCVD (OR 4.2, 95%CI 1.21–4.86, *p*=0.024). HCYS levels in the longitudinal observation did not vary.

**Conclusion:**

An increased CCVD risk, independent of other risk factors, is present in psoriatic patients with elevated HCYS. The HCYS level was not influenced by biological therapy in longitudinal observation. Further studies are needed to explore if elevated HCYS could serve as a marker of increased CCVD in any stage of psoriasis and if it should be included in classical screening strategies.

## 1. Introduction

Psoriasis is a multifactorial disease that generally involves the skin and joints. However, it is not considered just a skin disease or arthritis, as these symptoms are probably only a partial manifestation of wider systemic inflammation. Activation of nonspecific and specific immune factors with the development of inflammation plays a key role in the pathogenesis of psoriasis and is connected with a proatherosclerotic phenotype in animals [1]. Elevation of C-reactive protein, HCYS, inflammatory cytokines, and novel proteins (proprotein convertase, subtilisin/kexin type-9, angiopoietin-like protein 8, sortilin, and cholesteryl ester transfer proteins) [2] in psoriatic patients may contribute to cardio metabolic syndrome in humans with psoriasis. Potential mechanisms linking psoriasis and atherosclerosis are related to the inflammatory process itself, as well as other pathophysiological factors, such as metabolic disorders, lipid abnormalities, central obesity, and insulin resistance [3, 4]. However, epidemiological studies in psoriatic patients show variable results: from no increased cardiovascular events/disease [5, 6] to mild increase of myocardial infarction and increased myocardial infarction, stroke, and cardiovascular mortality [7, 8]. Detailed analysis shows that the severity and duration of psoriasis could play a role, and the risk of cardiovascular events could depend on them.

Hyperhomocysteine (HCYS) level is considered as a pathological orchestrator of proatherogenic reactions from dyslipidaemia to endothelial dysfunction, resulting in increased cardiovascular risk [9, 10]. HyperHCYS has also been linked to psoriasis [3, 11].

In this study, we sought to determine whether plasma HCYS levels in patients with moderate/severe psoriasis could be associated with an increased prevalence of cardio-cerebro vascular disease (CCVD)—stroke, myocardial infarction, and ischemic heart disease—and whether biological treatment, aimed at influencing the pathogenesis of the psoriasis, could affect HCYS levels in long-term observations.

## 2. Materials and Methods

### 2.1. Samples and Procedures

The study sample consisted of psoriatic patients enrolled for biological therapy at the Department of Dermatovenerology at Louis Pasteur University Hospital in Košice (UNLP) in the years 2009–2017. Patients came from the catchment area of the UNLP, which covers the Košice and Prešov regions, or almost one-third of the population of Slovakia. The inclusion criteria were as follows: diagnosis of moderate/severe form of psoriasis in patients who showed no improvement with traditional treatment, biological treatment as a planned main method of treatment, and age over 18 years. Participation in the study was voluntary, and patients were allowed to withdraw their agreement to participate at any time during the study. The study was approved by the ethical committee, and all patients completed an informed consent form.

The data used in this study come from real-life clinical practice. Patients were enrolled in the study prospectively depending on psoriasis severity when requiring biological therapy.

Demographic data (age of the patients at entrance to the study, gender, duration of the disorder, and family history of psoriasis), comorbid diagnoses, and treatment data were collected at the first visit. The Psoriatic Area Severity Index (PASI) was used as a standard tool for staging the severity and extent of psoriasis [12]. PASI, body mass index (BMI), and biochemical parameters were collected at the first visit and at regular intervals (every 3 months). The biochemical parameters involved HCYS, inflammatory markers (CRP: C-reactive protein; ESR: erythrocyte sedimentation rate), blood lipids (serum total cholesterol, LDL cholesterol, HDL cholesterol, and triacylglycerol), serum total bilirubin, and liver enzymes—alanine aminotransferase (ALT), gamma glutamyl transferase (GMT), alkaline phosphatase (ALP), and amylase (AMS). Fasting total HCYS plasma levels were assessed in a certified laboratory using a chemiluminescent microparticle immunoassay. Normal HCYS plasma levels ranged 5–15 *μ*mol/mL. The study population was divided into two groups based on the entrance level of HCYS: a group with normal and a group with elevated HCYS blood levels; the differences in biochemical and clinical parameters between the HCYS groups were statistically analysed.

**Figure 1:**
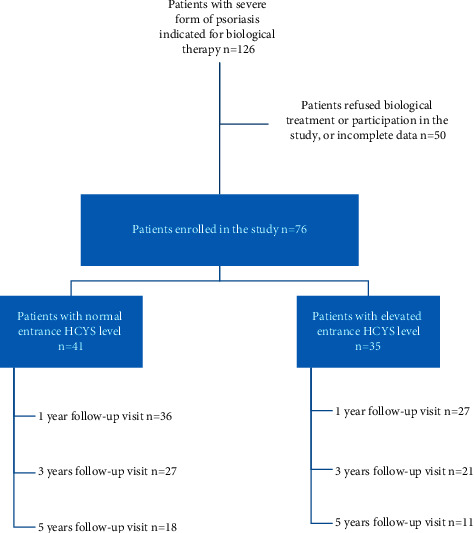
Flow chart of the number of participants in each step of the study.

In the long-term observation of HCYS plasma levels, PASI, and psoriasis treatment data were recorded at the entrance visit and during follow-up visits (periodically every 1, 3, 5, and 7 years). HCYS, PASI, and type of treatment were compared between the groups based on entrance HCYS levels (Figure 1).

### 2.2. Data Analysis

Descriptive statistics were performed initially for demographic data, clinical features, laboratory test results, and comorbid diagnoses. The effects of biochemical and clinical parameters on the likelihood of having elevated HCYS levels were tested using binary logistic regression. Pearson's Chi-squared test (chi^2^ test) and the Mann-Whitney *U*-test were used for continuous variables. Additionally, mixed method model analysis was used to test changes in HCYS plasma levels in time. Values of *p* < 0.05 were considered statistically significant. Data were analysed using the IBM SPSS 23.0 software package (IBM Corp., released 2015; IBM SPSS Statistics for Windows, Version 23.0. Armonk, NY: IBM Corp.).

## 3. Results

Seventy-six patients (mean age 46.7 years, SD 13.2 years, ranging from 18 years to 83 years, 19 females (25%) and 57 males (75%)) with a severe form of psoriasis (mean PASI 23.6, SD 7.7) were included in the study. Arterial hypertension (50%), psoriatic arthritis (49%), dyslipidaemia (49%), hepatopathy (45%), and diabetes mellitus type 2 (24%) were the most frequent comorbid diagnoses in psoriatic patients. Entrance HCYS levels were elevated above the normal range in 35 patients (46%) (Table 1).

### 3.1. Comparison of Demographic and Clinical Features, Laboratory Findings, and Comorbidities in Psoriatic Patients Based on the Entrance HCYS Plasma Level

Study groups with normal and elevated HCYS did not vary in age, gender, duration of psoriasis, PASI, BMI, history of psoriasis, smoking, or alcoholism.

Laboratory tested biochemical parameters did not reveal any statistically significant differences between the groups in systemic inflammatory markers, lipid profile, and liver enzymes.

Comorbid diagnoses, such as psoriatic arthritis, arterial hypertension, diabetes mellitus type 2, hepatopathy, dyslipidaemia, autoimmune thyroiditis, depression, inflammatory bowel disease, kidney or respiratory disease, glaucoma, and cataract, did not vary between the groups based on HCYS level. The only increased prevalence in patients with elevated HCYS level was found in the frequency of CCVD (OR 4.2, 95% CI 1.209–4.863, *p*=0.024) (Table 1).

### 3.2. Longitudinal Observation of HCYS Plasma Levels in the Groups Based on Entrance HCYS Level and Type of Treatment

**Figure 2:**
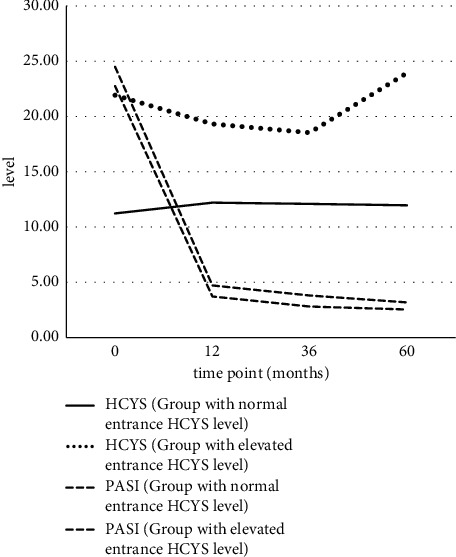
Trends in average HCYS blood levels and PASI scores at four time points in psoriatic patient subgroups with normal and elevated HCYS level. *Abbreviations.* HCYS: homocysteine; PASI: Psoriasis Area Severity Index.

Longitudinal observation of HCYS plasma levels showed no statistically significant changes in the first, third, and fifth year within the HCYS subgroups, as was shown by the mixed method model. The HCYS level did not differ significantly between time points. However, differences between groups were statistically significant in the HCYS level in all time points and statistically insignificant in PASI between groups in all time points, meaning that patients kept their normal or elevated HCYS level, respectively, during the observed period and improved their PASI with time almost identically. We could conclude that the HCYS level was not related to the psoriatic treatment, although PASI did improve during psoriatic treatment. Patients did not differ in the type of new set treatment: biological therapy (etanercept, adalimumab, and ustekinumab) with/without methotrexate. The drop-out rate in the seventh year was more than 50% in both groups, which is why the data were not included in the analysis. The reasons for dropping-out were personal, lack of compliance with treatment, or a shorter individual observational period (Table 2 and Figure 2).

## 4. Discussion

Psoriatic patients with elevated HCYS showed an increased prevalence of CCVD compared to patients with the same disease severity, age, and other comorbidities. The increased prevalence of CCVD was not associated with any other inflammatory and atherogenic laboratory abnormalities. In a longitudinal setting, the HCYS plasma level was not affected by biological therapy with/without methotrexate.

The relationship between psoriasis and HCYS has attracted attention for several years. Some studies looking at the relationship between psoriasis and atherosclerotic risk were criticized for their interpretation of the results, regardless of age or severity of the disease. Therefore, in our study, we decided to analyse cardiovascular risk in the group of patients with moderate/severe psoriasis who failed with traditional treatment options. We tried to identify the risk of cardiovascular comorbidity looking at HCYS as a generally considered marker of atherogenesis [9, 10]. Groups of psoriatic patients based on normal/abnormal HCYS levels did not differ in terms of age. We found that the patients with elevated HCYS had more frequent CCVD—stroke, myocardial infarction, and ischemic heart disease—while the groups did not vary in the frequency of independent cardiovascular risk factors, such as arterial hypertension, dyslipidaemia, or diabetes mellitus type 2. We did not reveal any differences between the groups in the prevalence of traditional risk factors associated with hyperHCYS (renal [13] and thyroid [14] dysfunction, past smoking [15], alcohol consumption [16], or BMI [17]). In the case of CCVD, some studies pointed at that coronary artery disease (CAD) was not just associated with elevated HCYS levels [18]; furthermore, HCYS levels correlated with the severity of CAD [18, 19]. In the present study, we can interpret our findings that hyperHCYS found in patients with moderate/severe psoriasis is connected with clinically significant atherosclerosis manifested by stroke, myocardial infarction, or coronary heart disease independent of other risk factors.

The concept of clustering of comorbid autoimmune disorders, for example, immune-inflammatory psoriasis and autoimmune thyroiditis, diabetes mellitus type 1, or inflammatory bowel disease, was not confirmed by our study. Psoriatic patients with elevated HCYS did show increased frequency of CCVD, but not autoimmune disorders, despite the fact that patients with psoriasis and CCVD could have the autoimmune mechanism accelerated due to the coincidence of psoriasis and CCVD.

No other inflammatory, lipid, and hepatic markers in our study were associated with an elevated HCYS level. Even if the association of elevated HCYS level with CCVD risk in psoriasis in our study population is suggestive and we excluded the role of traditional risk factors of hyper-HCYS, some of them could not be fully studied, as the study was designed as an observational study from daily routine practice: dietary factors, B vitamin deficiency, and genetic factors. On the other hand, it is not certain whether they could play a role in our results, if, for example, dietary intake of vitamin B on lowering HCYS was not recorded in patients with CCVD already present [20] or, similar to the presented study, the influence cardiovascular morbidity in chronic renal failure [21] and genetics was not studied in any other previous epidemiological study.

Management of moderate/severe psoriasis often requires systemic treatment (MTX, retinoids, cyclosporine A, dimethyl fumarate, and apremilast) or biologics. Biological therapy targets specific inflammatory pathways. We expected that this could affect not only the severity of the disease assessed by PASI (persistent therapeutic effect of treatment) but also the level of HCYS. Our results did not confirm that systemic treatment in the moderate/severe form of psoriasis would influence the HCYS level.

### 4.1. Strengths and Weaknesses of the Work

We consider acquiring of a relatively high number of specific psoriatic patients with moderate/severe form of psoriasis who failed with standard treatment methods to be a strong point of our study. We also would like to emphasize that, to the best our best of knowledge, the effects of HCYS levels on CCVD and other biochemical and clinical parameters have not been previously studied in a sample of psoriatic patients, and the potential relation of HCYS level with biological psoriasis treatment in a long-term observation has not been assessed. On the other hand, our study failed to retain the initial high number of patients during the whole observation period, which may undermine our findings from the long-term observations. We also want to mention that real-life clinical practice study has limited possibilities for measuring and examining further possibly relevant, but not clinically available parameters and confounders that might shed more light on the meaning of HCYS among psoriatic patients.

However, we consider the number of our patients to be relatively high, although the number was not enough to avoid the possible occurrence of a type II statistical error, meaning that some clinically relevant differences or associations may not appear as statistically significant. For confirmation or refusal of our findings about the role of HCYS among psoriatic patients, future research on a much larger number of psoriatic patients may be designed and special attention should be paid to the retention of patients in the sample during follow-ups.

## 5. Conclusion

Our findings suggest that elevated HCYS in patients with the moderate/severe form of psoriasis can identify those patients with an increased risk of severe CCVD. HCYS level is not influenced by biological treatment, and its longitudinal monitoring does not provide additional value for everyday routine practice. To evaluate the importance of HCYS testing in psoriasis, further studies are needed on whether HCYS can serve as a screening tool that with early intervention would reduce the risk of CCVD and whether it should be included in classical risk management strategies in psoriatic patients with any severity of the disorder.

## Figures and Tables

**Table 1:** Demographic characteristics, comorbidities, and laboratory tests of the psoriatic patients.

	Whole sample (*N* = 76) (100%)	Patient subgroups	Odds ratios (95% confidence intervals)	*p* value (binary logistic regression)	
		HCYS-normal level (*N* = 41) (53.9%), *M* = 11.23 (SD = 2.09)	HCYS-elevated level (*N* = 35) (46.1%), *M* = 21.92 (SD = 12.06)		
Demographics (categorical)	*N* (%)	*N* (%)	*N* (%)		
Gender (female vs. male)	19 (25.0%)/57 (75.0%)	11 (26.8%)/30 (73.2%)	8 (22.9%)/27 (77.1%)	0.808 (0.283–2.306)	0.690
Family history of PsO (positive)	34 (44.7%)	21 (51.2%)	13 (37.1%)	0.563 (0.224–1.411)	0.220
Demographics (continuous)	Mean (SD)	Mean (SD)	Mean (SD)		*p* value
PsO-duration (years)	20.62 (11.49)	21.76 (9.49)	19.29 (13.49)	0.981 (0.942–1.021)	0.367
Age (at time of examination)	46.70 (13.16)	44.59 (11.21)	49.17 (14.92)	1.028 (0.992–1.066)	0.131
PASI (input value)	23.55 (7.70)	22.75 (6.68)	24.50 (8.76)	1.031 (0.971–1.094)	0.325
BMI	31.51 (6.96)	30.09 (7.88)	32.92 (6.02)	1.067 (0.921–1.238)	0.388
Comorbidities (categorical) (positive vs. negative)	*N* (%)	*N* (%)	*N* (%)		*p* value
Smoking (yes)	34 (44.7%)	21 (51.2%)	13 (37.1%)	0.563 (0.224–1.411)	0.219
Alcoholism (yes)	24 (31.6%)	14 (34.1%)	10 (28.6%)	0.771 (0.290–2.049)	0.603
PsA	37 (48.7%)	23 (56.1%)	14 (40.0%)	0.522 (0.209–1.303)	0.164
Arterial hypertension	38 (50.0%)	18 (43.9%)	20 (57.1%)	1.704 (0.686–4.234)	0.251
CCVD	15 (19.7%)	4 (9.8%)	11 (31.4%)	4.240 (1.209–14.863)	0.024^*∗*^
DM II	18 (23.7)	10 (24.4%)	8 (22.9%)	0.919 (0.317–2.660)	0.876
Hepatopathy	32 (42.1)	15 (36.6%)	17 (48.6)	1.637 (0.654–4.101)	0.293
Dyslipidaemia	37 (48.7)	20 (48.8%)	17 (48.6%)	0.992 (0.402–2.445)	0.985
Autoimmune thyroiditis	4 (5.3%)	2 (4.9%)	2 (5.7%)	1.887 (0.297–12.005)	0.871
Depression	6 (7.9%)	1 (2.4%)	5 (14.3%)	3.284 (0.778–13.856)	0.105
IBD	3 (3.9%)	2 (4.9%)	1 (2.9%)	1.219 (0.162–9.141)	0.652
Kidney disease	3 (3.9%)	3 (7.3%)	0	n/a	0.102
Respiratory disease	6 (7.9%)	3 (7.3%)	3 (8.6%)	1.689 (0.351–8.131)	0.513
Glaucoma	2 (2.6%)	1 (2.4%)	1 (2.9%)	1.212 (0.073–20.131)	0.893
Cataract	3 (3.9%)	1 (2.4%)	2 (5.7%)	0.895 (0.186–4.308)	0.890
Lab tests (categorical)	*N* (%)	*N* (%)	*N* (%)		*p* value
ESR (elevated level)	42 (55.3%)	22 (53.7%)	20 (52.9%)	1.152 (0.464–2.856)	0.761
Lab tests (continuous)	Mean (SD)	Mean (SD)	Mean (SD)		*p* value
HCYS (*µ*mol/mL)	16.14 (8.91)	11.23 (2.09)	21.92 (12.06)	n/a	n/a
CRP (mg/l)	3.85 (4.37)	3.15 (3.60)	4.68 (5.08)	1.088 (0.967–1.224)	0.159
Cholesterol (mmol/L)	5.26 (1.27)	5.35 (1.02)	5.14 (1.52)	0.877 (0.607–1.267)	0.483
Triglycerides (mmol/L)	1.82 (1.22)	1.91 (1.13)	1.17 (1.33)	0.879 (0.592–1.303)	0.520
HDL cholesterol (mmol/L)	1.26 (0.39)	1.32 (0.42)	1.18 (0.35)	0.364 (0.092–1.435)	0.149
LDL cholesterol (mmol/L)	3.21 (0.98)	3.21 (0.91)	3.21 (1.07)	1.000 (0.611–1.638)	0.999
Bilirubin (*µ*mol/L)	12.34 (6.23)	11.12 (5.53)	13.78 (6.76)	1.076 (0.993–1.166)	0.073
ALT (*µ*kat/L)	0.53 (0.34)	0.49 (0.33)	0.58 (0.36)	2.076 (0.527–8.173)	0.296
GMT (*µ*kat/L)	0.69 (0.78)	0.55 (0.31)	0.85 (1.10)	2.668 (0.756–9.414)	0.127
ALP (*µ*kat/L)	1.31 (0.40)	1.26 (0.35)	1.36 (0.46)	1.861 (0.584–5.927)	0.293
AMS (*µ*kat/L)	0.92 (0.37)	0.95 (0.36)	0.88 (0.37)	0.601 (0.159–2.270)	0.452

*Abbreviations.* ALP: alkaline phosphatase; ALT: alanine aminotransferase; AMS: amylase; BMI: body mass index; CCVD: cardio-cerebrovascular diseases; CRP: C-reactive protein; DM II: diabetes mellitus type 2; ESR: erythrocyte sedimentation rate; GMT: gamma glutamyl transpeptidase; HCYS: homocysteine; HDL: high-density lipoprotein cholesterol; IBD: inflammatory bowel disease; LDL: low-density lipoprotein cholesterol; PASI: Psoriasis Area Severity Index; PsA: psoriatic arthritis; PsO: psoriasis. ^*∗*^*p* < 0.05. n/a: statistical analysis not applicable. Absolute and relative (%) prevalence or mean value with standard deviation (SD), respectively, presented for the whole sample and the HCYS subgroups. Subgroup difference or variable effect is presented as odds ratio (OR) with 95% confidence interval (CI) and its *p* value; statistical significance level is *p* < 0.05.

**Table 2:** Longitudinal observation of HCYS levels (*µ*mol/ml), PASI with type of therapy in groups based on the HCYS entrance level, difference tested by *U*-test or chi^2^ test.

		*Patient subgroups*		*p* value
		HCYS-normal level	HCYS-elevated level	
Entrance visit	*N*	41	35	
	HCYS (mean ± SD)	11.93 ± 2.10	21.06 ± 11.12	^*∗∗∗*^
	PASI (mean ± SD)	22.75 ± 6.68	24.5 ± 8.76	0.546
	Therapy (categorical)	N (%)	N (%)	
	Biologics: Etanercept Adalimumab Ustekinumab	22 (53.7%) 14 (34.1%) 5 (12.2%)	20 57.1%) 8 (22.9%) 7 (20.0%)	0.449^a^
	Biologics and MTX	10 (24.4%)	8 (22.9%)	0.550^a^

1-year follow-up	*N*	36	27	
	HCYS (mean ± SD)	12.21 ± 4.1	19.33 ± 11.31	^*∗∗∗*^^b^
	PASI (mean ± SD)	3.72 ± 2.96	4.72 ± 5.18	0.278^b^

3-year follow-up	*N*	27	21	
	HCYS (mean ± SD)	12.1 ± 3.94	18.54 ± 12.58	^*∗∗*^^b^
	PASI (mean ± SD)	2.8 ± 1.74	3.81 ± 2.43	0.168^b^

5-year follow-up	*N*	18	11	
	HCYS (mean ± SD)	11.97 ± 2.34	23.93 ± 20.75	^*∗*^^b^
	PASI (mean ± SD)	2.54 ± 2.1	3.18 ± 2.02	0.596^b^

*Abbreviations.* HCYS: homocysteine; MTX: methotrexate; PASI: Psoriasis Area Severity Index. ^a^Pearson's chi-squared test. ^b^Mann–Whitney *U*-test. ^*∗*^*p* < 0.05; ^*∗∗∗*^*p* < 0.001.

## Data Availability

Datasets are available upon request.
